# Design of the 18-year follow-up of the Danish COPSAC2000 birth cohort

**DOI:** 10.1136/bmjpo-2024-002634

**Published:** 2024-08-30

**Authors:** Trine Mølbæk-Engbjerg, Nilo Vahman, Marianne Mikkelsen, Nadia Rahman Fink, Emil Dalgaard Christensen, Nicklas Brustad, Lærke Sass, Hedda Løvenhøj, Katrine Strandberg-Larsen, Jonathan Groot, Anne-Marie Nybo Andersen, Rebecca Vinding, Ann-Marie Malby Schoos, Jakob Stokholm, Klaus Bønnelykke, Bo Chawes

**Affiliations:** 1 Department of Pediatrics, Copenhagen Prospective Studies on Asthma in Childhood, Slagelse Hospital, Slagelse, Denmark; 2 Copenhagen Prospective Studies on Asthma in Childhood, Herlev and Gentofte Hospital, University of Copenhagen, Copenhagen, Denmark; 3 Department of Public Health, University of Copenhagen Section of Epidemiology, Copenhagen, Denmark; 4 Department of Clinical Medicine, University of Copenhagen Faculty of Health and Medical Sciences, Copenhagen, Denmark

**Keywords:** Adolescent Health, Data Collection, Obesity, Noncommunicable Diseases, Child Health

## Abstract

**Background:**

Atopic diseases, obesity and neuropsychiatric disorders are lifestyle-related and environmental-related chronic inflammatory disorders, and the incidences have increased in the last years.

**Objective:**

To outline the design of the 18-year follow-up of the Copenhagen Prospective Study on Asthma in Childhood (COPSAC_2000_) birth cohort, where risk factors of atopic diseases, obesity and neuropsychiatric disorders are identified through extensive characterisation of the environment, along with deep clinical phenotyping and biosampling for omics profiling.

**Methods:**

COPSAC_2000_ is a Danish prospective clinical birth cohort study of 411 children born to mothers with asthma who were enrolled at 1 month of age and closely followed at the COPSAC clinical research unit through childhood for the development of atopic diseases. At the 18-year follow-up visit, biomaterial (hair, blood, urine, faeces, throat, and skin swabs, nasal lining fluid and scraping, and hypopharyngeal aspirates) and extensive information on environmental exposures and risk behaviours were collected along with deep metabolic characterisation and multiorgan investigations including anthropometrics, heart, lungs, kidneys, intestines, bones, muscles and skin. Neuropsychiatric diagnoses were captured from medical records and registers accompanied by electronic questionnaires on behavioural traits and psychopathology.

**Results:**

A total of 370 (90%) of the 411 cohort participants completed the 18-year visit. Of these, 25.1% had asthma, 23.4% had a body mass index >25 kg/m^2^ and 16.8% had a psychiatric diagnosis in childhood. A total of 68.7% drank alcohol monthly, and when drinking, 22.2% drank >10 units. Of the participants, 31.4% were currently smoking, and of these, 24.1% smoked daily. A total of 23.8% had tried taking drugs, and 19.7% reported having done self-destructive behaviour. The mean screen time per day was 6.0 hours.

**Conclusion:**

This huge dataset on health and habits, exposures, metabolism, multiorgan assessments and biosamples from COPSAC_2000_ by age 18 provides a unique opportunity to explore risk factors and underlying mechanisms of atopic disease and other lifestyle-related, non-communicable diseases such as obesity and neuropsychiatric disorders, which are highly prevalent in the community and our cohort.

**WHAT IS ALREADY KNOWN ON THIS TOPIC**

- Atopic diseases, obesity and neuropsychiatric disorders have increased in incidence in recent years.

**WHAT THIS STUDY ADDS**

- Detailed data on health and habits, exposures, metabolism, multiorgan assessments and biosamples from Copenhagen Prospective Study on Asthma in Childhood by age 18.

**HOW THIS STUDY MIGHT AFFECT RESEARCH, PRACTICE OR POLICY**

- The data provide a unique opportunity to explore risk factors and underlying mechanisms of atopic disease and other lifestyle-related, non-communicable diseases.

## Introduction

 Atopic diseases such as asthma, atopic dermatitis and allergic rhinitis are some of the most common chronic, inflammatory diseases in children and show an increasing prevalence.^1 2^ At the same time, an overlap between atopic and other non-communicable, lifestyle-related diseases, such as obesity and neuropsychiatric disorders, has been observed, proposing environment or lifestyle-induced mechanisms in early life as a common denominator.^3^,^8^ Children with asthma have an increased risk of developing depression and anxiety,^9^ and more recently, a link to attention deficit hyperactivity disorder (ADHD) was observed.^10^ Also, asthma is associated with metabolic syndrome and obesity, and speculations on a link between multiple prenatal exposures causing low-grade systemic inflammation have been proposed.^11^,^13^ Our main hypotheses are as follows: (1) Asthma and other chronic non-communicable inflammatory disorders such as obesity and neuropsychiatric disorders are programmed in early life and may share environmental risk factors; (2) Dysregulation of the immune system in early life can lead to chronic inflammatory diseases such as asthma, obesity and neuropsychiatric disorders; (3) There is an overlap between 18 years with asthma, obesity and neuropsychiatric disorders and (4) An individual’s capacity to metabolise nutrients is associated with a risk of asthma, obesity and neuropsychiatric disorders.

The Copenhagen Prospective Study on Asthma in Childhood (COPSAC_2000_) is a birth cohort study of children at risk of asthma designed to assess gene–environment interactions and early-life exposures that can be modified to prevent the development of atopic diseases. This paper aims to outline the hypotheses and the planned analyses and provide details of the 18-year follow-up visit of the COPSAC_2000_ cohort, including an extensive metabolic and multiorgan assessment and biobanking for omics analyses. This valuable information and material provide a unique possibility for studying risk factors and underlying mechanisms in the origins of atopic disease, obesity, neurodevelopmental disorders and their overlap.

## Methods

### Study population

**Figure 1:**
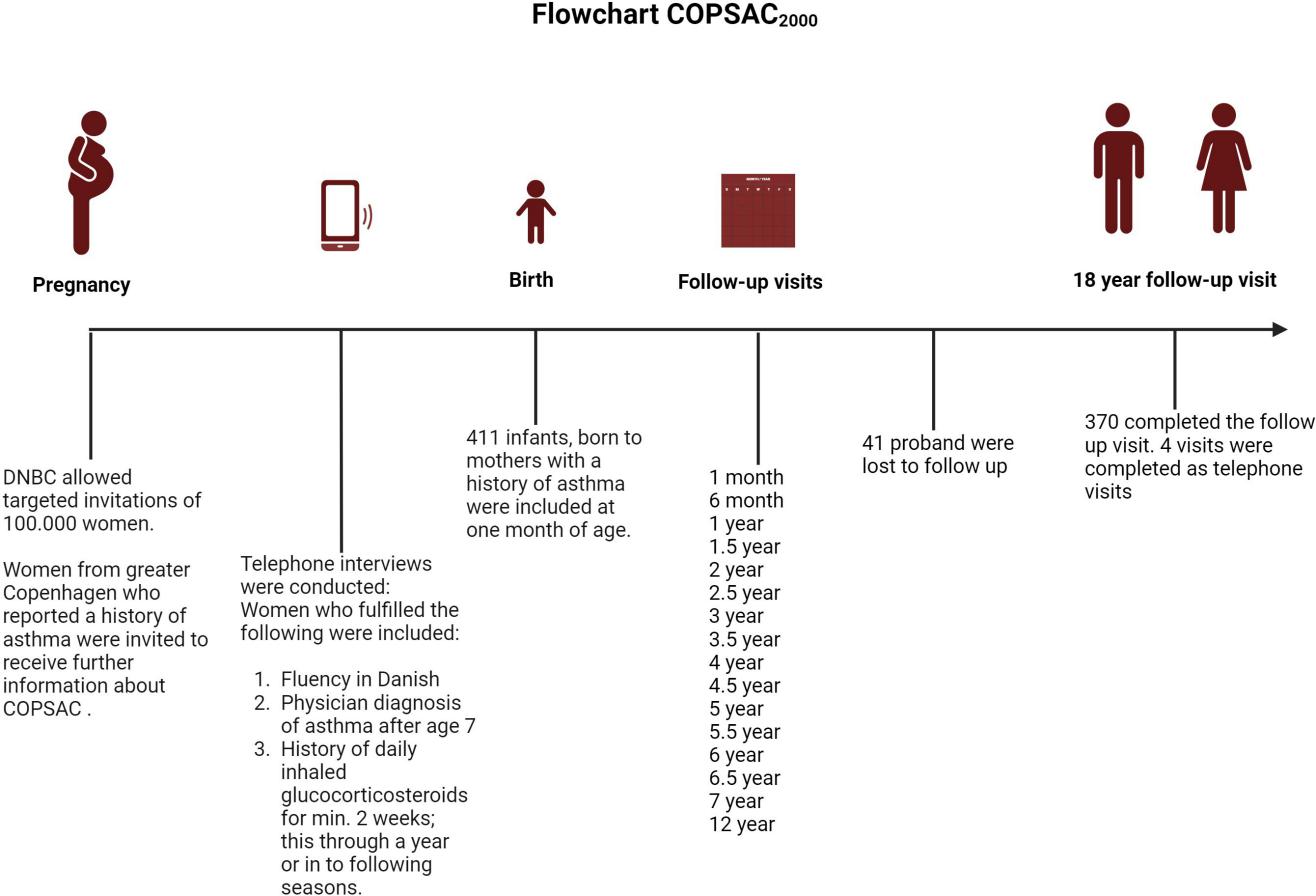
Flowchart illustration of the inclusion process of the COPSAC_2000_ cohort, and the follow-up visits until the 18-year follow-up visit. Created in Biorender.

The COPSAC_2000_ is a single-centre clinical, prospective, mother–child cohort study of 411 individuals born to mothers with asthma who were recruited during pregnancy from the Danish National Birth Cohort (DNBC). The children were included at one month of age and visited the COPSAC research unit at scheduled visits every 6 months until age 7 and again at ages 12 and 18. Further, the children visited the research unit on the occurrence of any acute airway or skin symptoms. The recruitment and baseline characteristics of COPSAC_2000_ are previously described in detail^1^ (see figures1^3^).

**Figure 2:**
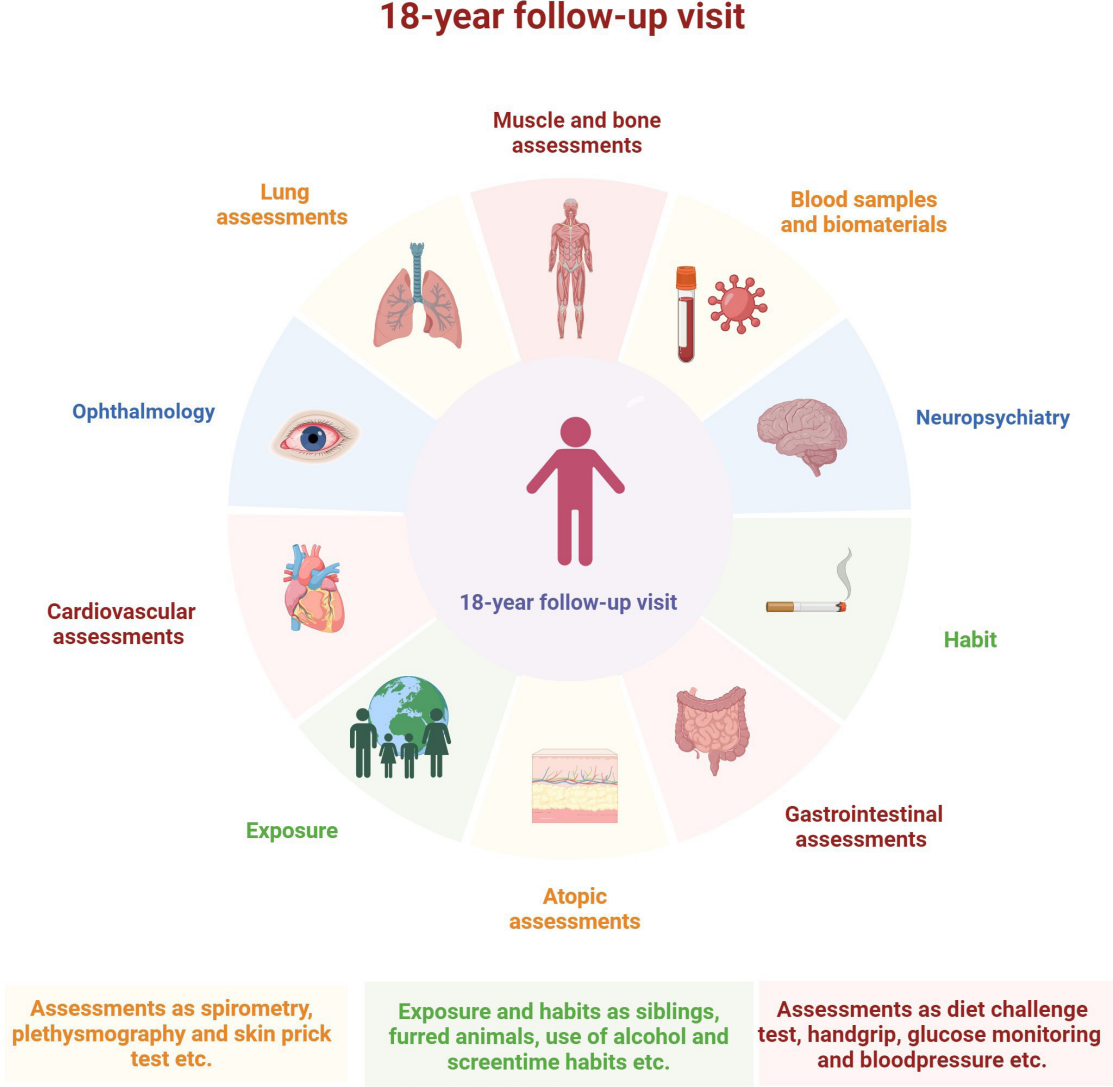
Illustration of the assessments described at the 18-year visit. Created in Biorender.

**Figure 3:**
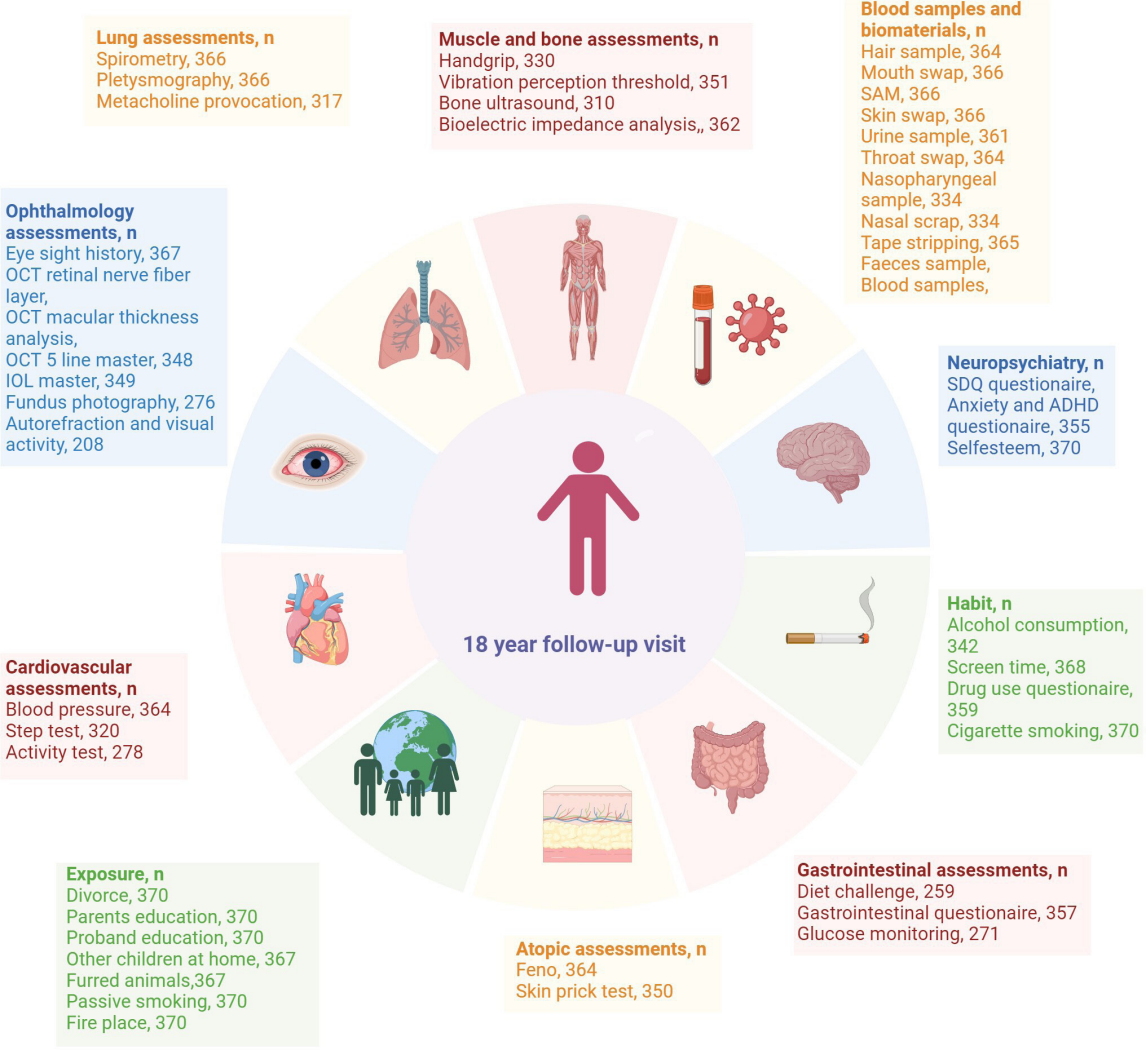
Illustration of the assessments described at the 18-year visit. Created in Biorender. ADHD, attention deficit hyperactivity disorder; SDQ, Strengths and Difficulties Questionnaire; OCT, optical coherence tomography; IOL, Intraocular Lens.

Initial recruitment into and participation in DNBC, which is a large general population birth cohort, has been previously described in detail. ^14^ In this study, we compare available and comparable baseline characteristics from a large sample of complete observations from the 18-year follow-up of DNBC with the baseline characteristics of COPSAC_2000_ at age 18.

### Environmental exposures and habits

Information on educational level, other children living in the home, exposure to pets, passive smoking and fireplaces was collected. We registered personal patterns of substance use, such as alcohol, tobacco and drug use. The participants were also asked about self-destructive behaviour. Further, the participants were interviewed about screen time habits, characterised as time spent on a screen in their spare time during the week (Monday to Thursday) and on weekends (Friday to Sunday).

### Medicine and general medical history

The National Medicine Registry was checked for prescriptions redeemed up to 2 years before the visit date, compliance and duration of intake. We registered all medical diagnoses at the visit through interviews with the families. Hospital records were checked, and all contacts with the healthcare system were registered in the database. Any missed medical diagnoses were appended retrospectively at the visit and confirmed through the interview with the participant.

### Atopic disease assessments

A physical examination was conducted at the visit, primarily focused on skin details, diagnosing eczema in subjects with skin issues following Hanifin and Rajka criteria.^15^Asthma was solely diagnosed by the COPSAC physicians. Children with typical symptoms of asthma were defined as having asthma if at least two of the following criteria were fulfilled: (1) Obstructive lung function with forced expiratory volume in 1 s (FEV1)<80% and/or FEV_1_/forced vital capacity <80% measured by spirometry and/or specific airway resistance (sRaw)>1.6 kPa/s; (2) Bronchodilator reversibility: change of FEV1 c12% or sRaw ≥30% from baseline after inhalation of short-acting beta-2-agonist; (3) Airway inflammation: fractional exhaled nitric oxide (FeNO) ≥25 ppb and/or (4) Bronchial hyper-responsiveness to methacholine (drop in FEV1 ≥20% from baseline by a dose of methacholine ≤8 mg/mL) and/or exercise (≥10% decrease in FEV1 from baseline). Remission was defined as no symptoms or asthma treatment for 1 year. ^16 17^

Allergic rhinitis diagnosis was based on allergic sensitisation and a corresponding history of sneezing, blocked, runny or itching nose on relevant exposure in the past 12 months in periods without a concurrent cold.^18^

### Lung function tests and allergic sensitisation

Lung function was measured by spirometry (MasterScope Pneumoscreen, Erich Jäeger, Würzburg, Germany),^19^ and sRaw was assessed by whole-body plethysmography (MasterScope Bodybox, Erich Jäeger, Würzburg, Germany).^20^ FeNO was measured using the Denox 88 version 88 DX0256, Switzerland.^21^ Bronchial reactivity was measured by methacholine provocation using the APS Pro, CareFusion 234, Germany.^22^ Allergic sensitisation to common inhalants and food allergens was diagnosed based on a skin prick test and measurements of specific IgE^18^ .

### Neuropsychiatric diagnosis and mental health

We collected psychiatric diagnoses from the medical records, including any psychiatric diagnosis, former and present. The diagnoses were divided into subgroups: ADHD, autism spectrum disorder, anxiety, depression, eating disorder, mental retardation, developmental disorder, personality disorder, schizophrenia, post-traumatic stress disorder, obsessive–compulsive disorder and others.

During the visit, three electronic questionnaires on behavioural traits and psychopathology were completed: the Strengths and Difficulties Questionnaire, the Adult ADHD Self Report Scale and the Depression, Anxiety and Stress Scale-21.

Self-rated social position, self-esteem, was registered on a scale from 1 to 5, where 1 represented a subjective feeling of being unpopular or very low in the social hierarchy and 5 represented a subjective feeling of being the most popular and socially accepted person in the group of peers.

### Metabolic assessments

We performed a standardised metabolic meal challenge after fasting. First, we took blood and urine samples at baseline; whereafter, the participant drank a smoothie made of palm oil, dextrose, skimmed milk powder and water. Continuously with the meal challenge test, we took blood and urine samples consecutively to observe the decomposition of the products using metabolomics assessments. A flash glucose monitoring (Freestyle Libre) was performed by applying a subcutaneous sensor to the posterolateral part of the non-dominant upper arm. The sensor measured glucose levels every minute, summarising measurements every 15 min for 2 weeks. We simultaneously made a food photo diary for 14 days, where the participants took pictures of all intakes of food and beverages except for water. We measured height and weight,^1^ and blood pressure was assessed in a relaxed sitting position, registering three consecutive measurements. We assessed spontaneous physical activity using an omnidirectional accelerometer (ActiGraph wGT3X+Actilife) on the non-dominant wrist continuously for the following 2 weeks, assessing activity during the day and sleep.^23 24^

### Blood samples

At the beginning of the visit, we collected fasting blood samples, which were analysed for haematology, infection status, immunology, endocrinology/metabolic panel, inflammation markers, coagulation, proteins, vitamins, kidney and liver function, and sex hormones.

### Immunological assessments

Blood samples were analysed for multiple immunological outcomes such as C reactive protein (CRP), IgA, IgG, IgM, IgE, transglutaminase IgA, and IgG, and a broad selection of antibodies such as ANA, ANCA, SR and CCP.

We also assessed airway immune profiles by quantifying 18 cytokines in upper airway fluid collected with filter paper strips as previously detailed.^24^

### Ophthalmological outcomes

We measured uncorrected visual acuity, refractive error and best-corrected visual acuity using an automated refractor (ARK-1s, NIDEK CO. LTD, Hiroishi-cho, Japan). The axial length of the eyes was measured with an optical biometer (IOLMaster, Carl Zeiss Meditec AG, Jena, Germany). We obtained a three-dimensional image of the macula and the optic disk in both eyes through optical coherence tomography (Cirrus HD-OCT Model 4000 (Carl Zeiss Meditec AG, Jena, Germany).^25^ Finally, a colour fundus photography was performed using a non-mydriatic retinal camera (TRC-NW7SF Mark II, Topcon, Tokyo, Japan). We recorded any parent-reported ophthalmological diagnoses during childhood. Additionally, the participant was interviewed regarding glasses or contact lenses, prescribed power and a history of squinting at any time during childhood.

### Bone and muscle assessments

We measured body Composition Analysis by Bioelectrical Impedance Analysis using a Tanita scale (TANITA MC-780MA) to analyse height-dependent muscle mass rating, skeletal muscle mass, fat percentage, and distribution, body mass index (BMI), body water distribution, and basal metabolic rate. A Saehan DHD-1 digital hand dynamometer was used to measure handgrip strength.^26^ We measured the density of the bones with an ultrasound of the non-dominant hand (IGEA DBM Sonic BP, Bone Profiler, Model: BP01). The vibration perception threshold was measured under the feet using a handheld biothesiometer (G&G MediCare). We assessed the participant’s maximum oxygen consumption (VO2max in L/min) using The Danish Steptest^25 27^ to estimate cardiorespiratory fitness.

### Gastrointestinal assessment

During the visit, the participants completed electronic questionnaires regarding gastrointestinal symptoms and the Bristol stool chart.^27^

### Biobank materials

At the visit, we collected multiple biosamples. These included a throat swab for virus detection, a mouth swab for bacteria, a nasopharyngeal sample, a nasal scrap for mRNA, upper airway lining fluid collected using a synthetic absorptive matrix for cytokines, tape stripping for collecting skin layer cells, a skin swab from the axil and antebrachium, a hair sample, a urine sample and a faecal sample for 16s rRNA sequencing and gut metabolomics.

### Omics analysis

We are currently planning the following omics analysis on the biobank materials collected at the 18-year follow-up visit: (1) Plasma metabolomics, 0–4 hours, evaluated before and after a nutritional challenge test including 1H-NMR metabolomics measured at eight different times over 4 hours; (2) Plasma hormones (GIP, GLP1, Insulin, C peptide and Glucagon), 0–4 hours, evaluated before and after a nutritional challenge test measured at eight different times over 4 hours; (3) Stool metagenomics (microbiome); (4) Stool metabolomics (Based on NMR) and (5) Stool viromics.

### Data management and statistics

We made data collection following Good Clinical Practice guidelines. We obtained all information at the 18-year follow-up visit through personal interviews done by medical doctors and research assistants with paediatric training, using predefined questions and closed questions response categories. Standard operating procedures were predefined for all study and database registration procedures. We collected data online during the visit. We saved any editing and changes of the data in an audit trail in the database documents. We double-checked the database against source data by an external person. We used data from participants who completed the 18-year visit, and missing values were treated as missing observations.

Data were analysed descriptively with counts N (%), mean (±SD) and median (IQR). Additionally, we conducted a t-test or X^2^ test to assess differences between genders. In cases without normal distribution, the Wilcoxon test was used. Data processing was conducted with R (R Foundation for Statistical Computing, R V.4.0.3 (2020-10-10), Vienna, Austria).

### Planned analyses

First, we plan to investigate the association between environmental risk factors and risk behaviours and asthma, obesity, and neuropsychiatric disorders at age 18 using univariate and multivariate logistic regression models. The overlap between asthma, obesity and neuropsychiatric disorders will be analysed using logistic regression and visualised with Venn diagrams.

Second, we plan to investigate the relationship between the immunological assessments cross-sectionally at age 18 years in relation to asthma, obesity, and neuropsychiatric disorders and longitudinally through childhood using latent class trajectories with multiple assessments from early childhood with random effects incorporated.

Third, we plan to analyse the association between the functional metabolism captured by changes in plasma metabolite levels during the standardised meal challenge using data-driven pricipal component analysis (PCA), time series analysis and supervised PLS-DA models (Partisal Least Squares Discriminant Analysis). In these models, we will integrate plasma hormone levels, gut metagenomics and stool metabolomics.

## Results

### Demographics

A total of 370 (90%) of the 411 children in the cohort completed the 18-year visit. The mean age was 17.7 years, with a maximum of 20.7 years and a minimum of 17.0 years; 49.4% were males and 96.6% were Caucasians. A total of 80% had started high school or similar. At home, 58.9% had younger children, 23.5% had older children and 38.4% had divorced parents. The cohort exhibited an equal distribution between rural and urban living environments, with 53% residing in rural areas and 47% in urban areas (table 1).

**Table 1:** Demographics

Cohort	DNBC*	COPSAC2000			
	All N (%)	All N (%)	Female N (%)	Male N (%)	P value†
Gender	44 485 (58.6 female)	370 (90)	188 (50.8)	182 (49.2)	0.35
Parents’ divorce	13 394 (30.1)	142 (38.4)	74 (39.4)	68 (37.4)	0.77
Race (Caucasian)	N/A	355 (96.0)	183 (97.3)	172 (95.0)	0.34
Proband education					
Elementary school, completed	N/A	357 (96.5)	182 (96.8)	175 (96.2)	1.00
College, started	35 280 (83.6)	288 (77.8)	155 (82.4)	133 (73.1)	0.07
Tradesman, started	4331 (10.3)	38 (10.3)	9 (4.8)	29 (15.9)	**<0.01**
University, started	361 (0.9)	1 (0.3)	–	1 (0.51)	–
Parents education, completed					
Elementary school, mother	N/A	13 (3.51)	8 (4.26)	5 (2.74)	0.41
Elementary school, father	N/A	17 (4.60)	11 (5.85)	6 (3.30)	0.23
High school, mother	N/A	6 (1.62)	6 (3.19)	–	–
High school, father	N/A	7 (1.89)	5 (2.66)	2 (1.10)	0.26
Tradesman, mother	N/A	94 (25.4)	39 (20.7)	55 (30.2)	0.10
Tradesman, father	N/A	102 (27.6)	47 (25.0)	55 (30.2)	0.43
University, medium long, mother	N/A	125 (33.8)	62 (33.0)	63 (34.6)	0.93
University, medium long, father	N/A	70 (18.9)	35 (18.6)	35 (19.2)	1.00
University, long, mother	N/A	69 (18.6)	41 (21.8)	28 (15.4)	0.12
University, long, father	N/A	82 (22.2)	40 (21.3)	42 (23.1)	0.83
Fireplace					
1–100 days	N/A	137 (37)	63 (33.5)	74 (40.7)	0.20
>100 days	N/A	38 (10.3)	18 (9.6)	20 (11.0)	0.53
Furred animals					
Cats, yes	N/A	100 (27.0)	52 (27.7)	48 (26.4)	0.87
Dogs, yes	N/A	181 (48.9)	96 (51.1)	85 (46.7)	0.46
Other children younger, yes	N/A	218 (58.9)	105 (55.9)	113 (62.1)	0.47
Other children older, yes	N/A	87 (23.5)	46 (24.5)	41 (22.5)	0.89
Current smoking	9639 (21.7)	116 (31.4)	58 (30.9)	58 (31.9)	0.38
Every day‡	3335 (34.6)	28 (24.1)	15 (25.9)	13 (22.4)	0.71
Min ones every week‡	2193 (22.8)	37 (31.9)	15 (25.9)	22 (37.9)	0.25
Min ones every month‡	4111 (42.7)	51 (44.0)	28 (48.2)	23 (39.7)	0.48
Number of cigarettes smoked in the last 4 weeks§					
1–4 per day	2879 (52.1)	82 (80.4)	36 (73.5)	46 (86.8)	0.35
5–9 per day	1326 (24)	11 (10.8)	9 (18.4)	2 (3.8)	0.08
>9 per day	1189 (21.5)	9 (8.8)	4 (8.1)	5 (9.4)	0.74
Passive smoking					
1–100 days per year	N/A	116 (31.4)	58 (30.9)	58 (31.9)	0.92
>100 days per year	N/A	110 (29.7)	61 (32.4)	49 (26.9)	0.29
Drug use ever	14 673 (33)	87 (23.8)	31 (16.8)	56 (30.8)	0.69
Hash/pot	13 907 (94.8)	60 (70.0)	23 (74.2)	37 (66.1)	0.06
Cocaine	1804 (12.3)	22 (25.3)	8 (25.8)	14 (25.0)	0.26
Laughing gas (NO)	1464 (10)	18 (20.7)	3 (9.7)	15 (26.8)	**0.01**
Ecstasy/MDMA/fantasy	1317 (9)	12 (13.9)	5 (16.1)	7 (12.5)	0.75
Others¶	2979 (20.3)	34 (39.1)	11 (35.5)	23 (41.1)	**0.04**
Alcohol; drinking frequency					
Never	3273 (7.4)	3 (1.0)	–	3 (1.6)	0.14
Max once per month	10 017 (22.5)	89 (24.1)	49 (26.1)	40 (22.0)	0.46
>1 per month	21 195 (70.1)	253 (68.4)	124 (66.0)	129 (70.9)	0.46
Alcohol; units					
1–4	11 853 (28.8)	88 (23.8)	51 (27.1)	37 (20.3)	0.26
5–9	21 952 (53.3)	172 (46.5)	93 (49.5)	79 (43.4)	0.41
>10	7407 (18)	82 (22.2)	29 (15.4)	53 (29.1)	0.91
BMI<18.5 (%) underweight	4395 (9.9)	32 (8.7)	17 (9.20)	15 (8.2)	0.72
BMI 18.5–24.99 (%) normal weight	32 336 (72.7)	249 (67.8)	121 (65.4)	128 (70.3)	0.66
BMI 25–30 (%) overweight	5770 (13)	57 (15.5)	31 (16.8)	26 (14.3)	0.51
BMI>30 (%) obese	1984 (4.5)	29 (7.9)	16 (8.7)	13 (7.1)	0.58
Screen time					
Total screen time hours, median (IQR)	N/A	6.0 (4.5–7.0)	5.90 (4.5–6.5)	6.7 (5.0–7.9	**<0.01**
Asthma, current	N/A	93 (25.1)	46 (24.4)	47 (25.8)	0.85
Self-injury	N/A	73 (21)	56 (31.1)	17 (9.9)	0.92
Self-esteem all (mean/SD)	N/A	3.58/0.80			
1		5 (1.35)	3 (3.23)	2 (1.10)	1.00
2		18 (4.86)	12 (12.9)	6 (3.30)	0.26
3		142 (38.4)	81 (87.1)	61 (33.5)	0.08
4		162 (43.8)	77 (82.80)	85 (46.7)	0.29
5		40 (10.8)	14 (15.1)	26 (14.3)	**0.05**
Neuropsychiatric disorder ever	N/A	62 (16.8)	30 (16.0)	32 (17.6)	0.80

Bold values represent p-values at or below the significance level of 0.05.

* Absolute and relative frequencies for a sample of complete response observations from the 18-year DNBC follow-up.

† t-test comparing significance between the gender.

‡ Only for current smokers.

§ Only for current smokers. Data are available from 102 probands.

¶ Amphetamine, sedatives, LSD, opiates, euphoric mushrooms, lighter gas.

BMIbody mass indexCOPSAC2000Copenhagen Prospective Study on Asthma in ChildhoodDNBCDanish National Birth CohortLSDLysergic Acid DiethylamideMDMA3,4-Methylenedioxy-methamphetamineN/Anot available

### Environmental exposures and habits

In their homes, 27.0% lived with a cat and 48.9% with a dog. A total of 37.0% were exposed to a fireplace at home 1–100 days per year, and 10.3% were exposed >100 days per year. A total of 61.1% were exposed to passive smoking, whereas 29.7% were exposed >100 days per year. At age 18 years, 31.4% of the participants were currently smoking, and of these, 24.1% smoked daily. Among smokers, the distribution of how many cigarettes they smoked per day was: 1–4/day, 80.4%; 5–9/day, 10.8% and >9/day, 8.8%. Only 1% had never drunk alcohol, whereas 68.4% drank more than once per month. When drinking, 68.7% drank >4 units per event, and 22.2% drank >10 units per event. A total of 23.8% had tried taking drugs. Of those, 70% had tried hash, 25.3% cocaine, 20.7% laughing gas (nitric oxide), 13.9% had tried ecstasy (MDMA, 3,4-methylenedioxy-methamphetamine) and 39.1% had tried other types of drugs such as amphetamine, sedatives, LSD (Lyserg-syre-Diethylamid), opiates, euphoric mushrooms and lighter gas. There were only gender differences with respect to laughing gas (p=0.01) and other drugs (p=0.04), which had been tried by males more often than females.

The mean screen time per day was 6.0 hours and was higher for males vs females: 6.7 vs 5.9 hours (p<0.01). This assessment included using television, games and phones in leisure time (table 1).

### Atopic diagnosis

A total of 44.3% had an asthma diagnosis at some point in the first 18 years of life. By age 18, 25.1% were diagnosed with asthma, allergic rhinitis was diagnosed in 38.6% of the probands, and atopic dermatitis was diagnosed in 10.3% (table 2). More females than males had atopic dermatitis (p=0.05), whereas there was a trend of more allergic rhinitis among males (p=0.06) and no gender differences in the presence of asthma by age 18.

**Table 2:** Atopic assessments and diagnoses

	All N (%)	Females N (%)	Males N (%)	P value*
Spirometry, FEV1†				
FEV1, L<80%	35 (9.5)	11 (5.9)	24 (13.2)	**0.03**
FEV1, L<90%	173 (46.8)	93 (49.5)	80 (44.0)	0.31
sRaw				
sRaw kPa/s>1.6 (%)	28 (7.6)	12 (6.4)	16 (8.8)	0.52
Methacholine provocation, median PD20/µmol‡	225.40	186.2	298.6	**0.01**
% <cut-off off	17 (4.6)	8 (4.3)	9 (5.0)	
Feno>25 ppb§ (%)	104 (28.1)	40 (21.3)	64 (35.2)	**<0.01**
IgE total x 103 IU/L, median (IQR)§	80.0 (28.0–238.1)	69.4 (25.0–216.5)	103.9 (32.4–262.6)	0.06
Positive specific IgE any kU/L, N (%)§	192 (51.9)	80 (42.6)	112 (61.5)	**<0.01**
Positive specific IgE food kU/L, N (%)§	72 (19.5)	34 (18.1)	38 (20.9)	0.6
Positive specific IgE inhalation kU/L, N (%)§	188 (50.8)	78 (41.5)	110 (60.4)	**<0.01**
Positive SPT any (%)	176 (47.6)	75 (39.9)	101 (55.5)	**<0.01**
Positive SPT food (%)	28 (7.6)	11 (5.9)	17 (9.3)	0.23
Positive SPT inhalation (%)	175 (47.3)	74 (39.4)	101 (55.5)	**<0.01**
Asthma, current (%)	93 (25.1)	46 (24.4)	47 (25.8)	0.86
Asthma, ever (%)	164 (44.3)	77 (41.0)	87 (47.8)	0.44
Rhinitis, current (%)	143 (38.6)	62 (33.0)	81 (44.5)	0.06
Atopic dermatitis, current (%)	38 (10.3)	25 (13.3)	13 (7.14)	**0.05**

Bold values represent p-values at or below the significance level of 0.05.

* Significance between the genders is calculated with t-test. In causes of not normal distributed data, Wilcoxon test has been used.

† Calibrated for sex, height and age.

‡ PD20 is the median dose of methacholine that causes a 20% reduction in FEV1. The test is positive when a 20% reduction in FEV1 is coursed by a PD20 <8 µmol.

§ Normal range FeNO <25 ppb.

FeNOfractional exhaled nitric oxideFEV1forced expiratory volume in 1 sSPTskin prick testsRawspecific airway resistance

### Lung function and allergy tests

The lung function tests showed that 9.5% had an FEV1<80%, 46.8% had an FEV1<90% and 7.6% had increased airway resistance, that is, sRaw>1.6 kPa/s. An increased FENO>25 ppb was seen in 28.1% of the probands. The methacholine provocation test was positive in 4.6%, defined as a 20% reduction in FEV1 caused by a dose <8 µmol/mL.

The median (IQR) total IgE level was 80.0×10³ IU/L (28.0–238.1); 19.5% had elevated specific IgE (>0.35 ku/L) towards foods and 50.8% had elevated specific IgE towards aeroallergens. The skin prick test was positive for foods in 7.6% and aeroallergens in 47.3% of the participants (table 2). In general, males more often had adverse lung function and allergy tests compared with females (table 2).

### Anthropometrics, bone and muscle assessments

The mean (SD) BMI was 22.8 kg/m^2^ (0.39) for males and 23.3 kg/m^2^ (0.42) for females. Males and females had an equal BMI distribution, and there was no significant difference between the genders when testing for this (p>0.51) (table 1). At the visit, 97.8% completed the body composition analysis, and 89.2% had handgrip strength measured. Bone density was measured by ultrasound in 83.8% of the probands and vibration tests were performed in 94.9%.

### Metabolic assessments

At the visit, 70.0% completed the meal challenge, 73% had their blood glucose monitored and 74.1% fulfilled the activity test. Blood pressure was mostly measured in the normal range; only 0.5% of the males and 0.5% of the girls had abnormally high blood pressure (males: MAP>103 mm Hg; females: MAP>99 mm Hg.).

### Blood samples

Blood sample results are presented with their mean or median, normal reference interval and percentage of participants with values outside the normal range in online supplemental appendix, for example, mean plasma 25-hydroxyvitamin D was 64 nmol/L with 31.4% of the participants having a value below the normal range (<50 nmol/L) and mean LDL (low-density lipoprotein) cholesterol was 2.0 with 7% having values above the upper range (>3 mmol/L) (online supplemental tables S1–S3).

### Immunological outcomes

The mean plasma IgA was 1.49 g/L, with 3.1% of females placed below the normal range vs 4.5% of males and 2.5% of females placed above the normal range vs 2.6% for males. IgG had a mean value of 10, with 3.8% of females placed below the normal range vs 5.8% of males and 0.6% of females placed above the normal range vs 2.6% of males. The median CRP was 0.74, with an IQR of 1.7 (online supplemental tables S1 and S3).

### Ophthalmological outcomes

Out of 366 children, eyesight history was available for 99.2% and 38% had used glasses or contact lenses at some point, with a myopia/hyperopia ratio of 3:1.

### Biobank material

For 98.9% of the probands, we collected mouth swabs for bacteria and skin swabs. Throat swabs for viruses, SAM, tape stripping and a hair sample were collected in 98.6% of the probands. In 97.3% of the cases, we collected a urine sample, nasopharyngeal samples and nasal scrap for mRNA in 90.3% of the proband. Only 58.1% delivered a faeces sample.

## Discussion

### Primary findings

In this extensive investigation of environmental risk factors, risk behaviours and multiorgan assessments, we obtained highly granulated information about the health and habits of Danish adolescents. We found that approximately one-fourth had asthma, one-fourth had a BMI>25 kg/m^2^ and 16.8% had a neuropsychiatric diagnosis in childhood. In addition, more than two-thirds of the 18-year-old Danish adolescents drank alcohol more than once per month, and when drinking, more than one-fifth drank >10 units of alcohol. Further, almost one-fourth had tried taking drugs.

### Perspective

This huge dataset on health and habits, exposures, metabolism, multiorgan assessments and biosamples for omics profiling from the COPSAC_2000_ birth cohort by age 18 years provides strong data to explore risk factors and metabolic mechanisms behind atopic diseases and other lifestyle-related, non-communicable disorders such as obesity and neuropsychiatric diseases and the commonality between these disorders. The standardised meal challenge is a unique opportunity to study the relationship between an individual’s metabolic capacity and the risk of asthma, obesity and neuropsychiatric disorders. This could pave the path for dietary intervention studies to prevent and treat these common disorders. The presentation of this rich data source, biobank material and methods is also an invitation for collaborative efforts with other cohorts worldwide.

### Strengths and limitations

The major strength of this study is the multiple assessments from most organ systems and the excessive amount of information describing the environment and risk behaviours of the participants. Furthermore, multiple biosamples are collected and stored for future omics analysis. All objective measurements were done by trained research personnel strictly following standard operating procedures. The participants of the COPSAC_2000_ cohort have participated in such examinations repeatedly from birth and are therefore highly competent in performing tests and examinations, which assure a high completion rate.

It is also a major strength that all diagnoses of asthma, atopic dermatitis and allergic rhinitis were diagnosed and monitored at the COPSAC research unit and not by general practitioners or based on parent interviews or questionnaires. All diagnostic procedures followed standard operating procedures, ensuring standardised diagnosis, treatment and regular evaluation. Furthermore, all participants were born to mothers with asthma, which improves symptom recognition.

A limitation of the study is its external validity because of the high-risk nature of the cohort, which was also predominantly Caucasians. However, comparing the 18 years in the DNBC allows us to compare differences in environmental factors, risk behaviours, obesity and asthma prevalence in a population-based cohort. Furthermore, we collected psychiatric diagnoses from the medical records, including any psychiatric diagnosis, former and present. The diagnoses were divided into 12 subgroups to simplify the classification. This simplified classification could cause a less precise diagnosis.

## Conclusion

This huge dataset on health and habits, exposures, metabolism, multiorgan assessments and biosamples from COPSAC_2000_ by age 18 years provides a unique opportunity to explore risk factors and metabolic mechanisms underlying atopic disease and the commonality with other lifestyle-related, non-communicable disorders such as obesity, dyslipidaemia and neuropsychiatric diseases, which are highly prevalent in the community and our cohort.

## supplementary material

10.1136/bmjpo-2024-002634 online supplemental file 1

## Data Availability

No data are available. All data relevant to the study are included in the article or uploaded as online supplemental information.
