# Supplementary material for: Design of the 18-year follow-up of the Danish COPSAC2000 birth cohort
Source: BMJ Paediatr Open. 2024 Aug 30;8(1):e002634. doi: 10.1136/bmjpo-2024-002634 (PMC11367328; doi:10.1136/bmjpo-2024-002634)
Supplement: online supplemental file 1 [file bmjpo-8-1-s001.pdf]

SUPPLEMENTARY

TABLE S1

**Table S1: Blood samples**		
**Lever**	**Median (IQR)**	**% placed outside normal reference interval**
ALAT<br>F:10–45 U/L<br>M:10-70 U/L	25 (10.0)	F: Low: 0.6; High: 4.4<br>M: Low: 0.6; High: 4.6
ASAT<br>F: 15-35 U/L<br>M: 15-45 U/L	28 (9.0)	F: Low: 1.9; High: 10.8<br>M: High: 7.8
Amylase<br>10-65 U/L	78.5 (17.5)	Low: 2.35<br>High: 11.1
Alkaline phosphatase<br>35-105 U/L	74 (31.0)	Low: 10.4<br>High: 6.5
Bilirubin<br>5-25 µmol/L/ L	10 (6.0)	Low: 1.17<br>High: 19.1
GGT<br>F: 10-45 U/L<br>M: 10-80 U/L	17 (10.0)	F: Low: 3.2; High: 1.9<br>M: Low: 0.7; High 0.7
LDH<br>105 - 205 U/L	146 (28.2)	Low: 19.9<br>High: 3.2
Haptoglobin<br>0.35 – 1.85 g/L	0.97 (0.6)	Low: 4.2<br>High: 5.8
	**Mean (SD)**	**% placed outside normal reference interval**
Albumin<br>36-48 g/L	43.3 (2.9)	Low: 10.6<br>High: 13.3
Antitrypsin<br>0.97-1.68 g/L	1.35 (0.3)	Low: 5.11<br>High: 13.7

**Coagulation**	**Median (IQR)**	**% placed outside normal reference interval**
Antithrombin<br>0.80-1.20 × 10³ int.enh. / L	1.0 (0.1)	Low: 2.1<br>High: 3.3
INR<br><1,2	1.1 (0.1)	High: 0.6
Homocystein<br>< 10.0 µmol/L	10.1 (4.7)	High: 2.4
	**Mean (SD)**	**% placed outside normal reference interval**
APTT<br>20-29 s	30.5 (5.2)	Low: 5.9<br>High: 17.8
**Nephrology**	**Median (IQR)**	**% placed outside normal reference interval**
Creatinine kinase, CK<br>F: 50-150 U/L<br>M: 50-270 U/L	93 (82.0)	F: Low: 18.4; High: 12.0<br>M: Low: 0.6; High 16.1
Creatinine<br>F: 45-90 µmol/L<br>M: 60-105 µmol/L	68 (15.0)	F: High: 0.6<br>M: Low: 6.5; High 0.6
Carbamide<br>F: 2.6-6.4 mmol/L<br>M: 3.2 – 8.1 mmol/L	4.5 (1.7)	F: Low: 3.2; High: 1.3<br>M: Low: 5.8; High: 0.6
Phosphate<br>F: 0.76 – 1.41 mmol/L<br>M: 0.71 – 1.53 mmol/L	1.19 (0.2)	F: High: 6.3<br>M: High: 3.9
PSA<br>< 3,0 µg/L	0.4 (0.4)	-***
	**Mean (SD)**	**% placed outside normal reference interval**
Kalium<br>3.5 – 4.6 mmol/L	141 (1.7)	Low: 10.9<br>High: 0.3
Calcium-ion frit<br>1.18-1.32 mmol/L	1.24 (0.0)	Low: 1.0<br>High: 0.3

Sodium<br>137 - 145 mmol/L	1.24 (0.0)	Low: 4.4<br>High: 20.9
Urate<br>F: 0.16-0.35 mmol/L<br>M: 0.23-0.48 mmol/L	0.311 (0.1)	F: Low: 2.5; High: 4.4<br>M: Low: 0.6; High: 0.6
**Coronary enzymes**	**Median (IQR)**	**% placed outside normal reference interval**
Troponin ITNI<br>< 24 ng/L	7 (11.5)	High: 1.5
ProBNP<br>No reference level	56.5 (35.8)	-
**Vitamins**	**Median (IQR)**	**% placed outside normal reference interval**
B12 vitamin<br>200 - 600 pmol/L	351 (162.0)	Low: 5.8<br>High: 5.1
Folate<br>>8.6 nmol/L	11 (7.5)	Low: 29.7
	**Mean (SD)**	**% placed outside normal reference interval**
D-vitamin, P-25-OH-Vitamin D(D3+D2)<br>50 - 160 nmol/L	64 (42.0)	Low: 31.4<br>High: 0.3
Jern<br>9 - 34 µmol/L	16.6 (6.8)	Low: 8.6<br>High: 1.2
Magnesium<br>0.70 – 1.10 mmol/L	0.822 (0.1)	Low: 1.6
**Haematology**	**Median (IQR)**	**% placed outside normal reference interval**
Platelets<br>F: 165 - 400 x 10^9/L<br>M: 145 - 350 x 10^9/L	250 (68.0)	F: Low: 1.2; High: 1.9<br>M: High: 4.6
Ferritin<br>13-152 µg/L	38 (41.0)	Low: 11.9<br>High: 2.3
Erythrocyte vol. Spred.<br>No reference levels.	13 (0.0)	-

Reticulocytes<br>F: 39-139 x 10^9/L<br>M: 39-139 x 110^9/L	58 (22.0)	F: Low: 12.4<br>M: Low: 7.1; High 0.6
Eosinophilic<br>>0.5 x 10^9/L	0.2 (0.2)	High: 5.6
Basophilic<br>>0.2 x 10^9/L	0 (0.1)	High: 33.8
Atypical cells<br>No reference levels	0.1 (0.0)	-
Monocytes<br>0.2-0.8 x 10^9/L	0.4 (0.2)	Low: 0.3<br>High: 1.2
Neutrophiles<br>1.6-5.9 x 10^9/L	2.99 (1.5)	Low: 1.5<br>High: 25.4
Transferrin saturation<br>0.2-0.5	0.24 (0.1)	Low: 33.1<br>High: 3.2
Transferritin, massek.<br>F: 2.12-3.91 g/L<br>M: 2.12-3.3 g/L	2.66 (0.6)	F: Low: 3.2; High: 3.2<br>M: Low: 7.1; High: 3.9
CRP<br>< 9 mg/L	0.74 (1.7)	High: 3.7
	**Mean (SD)**	**% placed outside normal reference interval**
Haemoglobin<br>F: 7.3 – 9.5 mmol/L<br>M: 8.3 – 10.5 mmol/L	8.62 (0.8)	F: Low: 5.7<br>M: Low: 3.3
MCV<br>82-98 fL	85.9 (3.6)	Low: 10.9
Erytrocytter<br>F: 3.94-5.16 x 10^{12}/L<br>M: 4.25-5.71 x 10^{12}/L	4.73 (0.4)	F: Low: 2.5<br>M: Low: 1.3; High: 0.6
Erytrocytter vol.fr<br>F: 0.35-0.46<br>M: 0.4-0.5	0.41 (0.1)	F: Low: 3.8<br>M: Low: 7.8
Lymphocytes<br>1.0-3.5 x 10^9/L	1.91 (0.5)	Low: 0.9<br>High: 0.3

MCH<br>1.7-2.1 fmol	1.83 (0.1)	Low: 2.9<br>High: 0.3
MCHC<br>19.7-22.2 mmol /L	21.3 (0.6)	Low: 0.3<br>High: 5.1
Leucocytes<br>3.5-8.8 x 10^9/L	5.8 (2.1)	Low: 1.0<br>High: 6.1
**Sex hormones**	**Mean (SD)**	**% placed outside normal reference interval**
Prolactin<br>F: 25-600 x 10^{-3} IU/L<br>M: 40-400 x 10^{-3} IU/L	196 (91.5)	F: High: 1.9<br>M: High: 2.0
Estradiol<br>F: Not normal references for this age group<br>M: > 0.3 nmol/L	0.15 (0.1)	-***
FSH<br>F: Not normal references for this age group<br>M: 1.4-7.5 IU/L	4.05 (3.5)	F: -***<br>M: Low: 3.3; High: 6.6
Progesteron<br>F: Not normal references for this age group<br>M: 0.7-4.3 nmol/L	1.8 (1.1)	-***
Lutropin, LH<br>F: 1-53 IU/L<br>M: 1-7.5 IU/L	3.9 (2.6)	F: High: 0.6<br>M: -***
HCG**<br>< 3 IU/L	1 (2.0)	-***
**Metabolism**	**Mean (SD)**	**% placed outside normal reference interval**
Proinsulin<br>336-4113 pmol/L	427 (222.0)	Low: 27.4
Insulin<br>20.8 – 173.6 pmol/L	71 (56.0)	Low: 0.6<br>High: 7.4

Hemoglobin A1c, IFCC<br>< 48 mmol/mol	5.4 (0.6)	High: 0.3
HbA1C, middle glucose<br>< 7.7 mmol/L	32 (3.0)	High: 0.3
Glucose<br>4.2-6.3 mmol/L	5.1 (0.5)	High: 1.6
Cholesterol, VLDL<br>> 0.9 mmol/L	0.4 (0.2)	High: 2.6
Cholesterol, HDL<br>< 1 mmol/L	1.18 (0.3)	***-
Cholesterol, LDL<br>>3 mmol/L	2 (0.7)	High: 7.0
Cholesterol, total<br>> 5 mmol/L	3.7 (0.8)	High: 2.9
Triglyceride<br>> 2 mmol/L	0.88 (0.5)	High: 3.2
PTH, parathyrin<br>2-8.5 pmol/L	4.84 (2.5)	Low: 1.0<br>High: 5.1
TSH, Thyrotropin<br>0.4-4.8 x 10^{-3} IU/L	1.82 (1.2)	High: 3.6
**Immunology and antibodies**	**Mean (SD)**	**% placed outside normal reference interval**
IgA<br>F: 0.65-3.06 g/L<br>M: 0.63-3.21 g/L	1.49 (1.0)	F: Low: 3.1; High: 2.5<br>M: Low: 4.5; High: 2.6
IgG<br>F: 7.4-15.6 g/L<br>M: 7.1-15.3 g/L	10 (2.6)	F: Low: 3.8; High: 0.6<br>M: Low: 5.8; High: 2.6
IgM<br>F: 0.66-2.61 g/L<br>M: 0.5-1.94 g/L	1 (0.6)	F: Low: 8.2; High: 1.3<br>M: Low: 8.4; High: 2.3

TS1 1: Blood samples: Presentation of blood samples with mean values and number in % of values placed outside normal reference interval.

:

M=Male, F=Female

*Samples not collected, haemolysed, or too little material to run the analysis.

**24 probands with positive values.

*** All values are placed in the normal interval.

TABLE S2

**Table S2: Faeces**	**Median (IQR)**	**Negative (%)**	**% placed outside normal reference interval**
**F-Calprotectin**<br><50: IBD** implausible<br>50 – 200: Elevated, repetition recommended<br>>200: Elevated, IBD** likely	34 (48.0)	109 (27)	>200: 2.1<br>50-200: 9.6<br><50: 22.0

Table S2:
*Samples not collected or too little material to run the analysis.
**Inflammatory bowel disease
or too little material to run the analysis.

TABLE S3

**Table S3: Immunology and antibodies**	**Median (IQR)**
Smith's Antibody	2.0 (2.0)
Sedimentations-reaction, SR	7.0 (3.3)
Cykl.citrul.peptid antibody, CCP	1.0 (1.0)
Sjögrens syndrome A IgG	32.0 (0.0)
Sjögrens syndrome B IgG	1.0 (0.5)
Transglutaminase IgA	2.0 (7.5)
Transglutaminase IgG	1.0 (1.0)
rRNA 2'O-methyltransferase fibrillarin-anti (IgG)	1.3 (1.0)
Centromer B antibody IgG – CBAIgG	3.2 (1.0)
P-Histidin--tRNA-ligase(Jo 1) -antistof(IgG)	-
P-DNA (dobbel-stranded) -antibody(IgG)	4.9 (4.5)
P-Chromodomain-helicase-DNA-binding protein 4-antibody (IgG)	7.2 (9.0)
P-proliferating nucleus-antibody (IgG)	2.0 (0.0)
P-ACE (Peptidyl dipeptidase A)	51.2 (22.0)
P-Exosome component 10-antibody (IgG)	1.3 (0.3)
P-Connective tissue related disease antibody	0.3 (0.2)
Scleroderma IgG	1.7 (1.0)

P-Ribonucleo protein U1 (70 kDA+A+C) - antibody (IgG)	2.7 (1.0)
P-DNA-directed RNA polymerase III subunit RPC1-antibody (IgG)	3.0 (2.0)
P-Acidic ribosomal protein antibody (IgG)	1.4 (0.3)
Rheuma-factor IgM	20.0 (2.0)
