# Supplementary material for: Design of the 18-year follow-up of the Danish COPSAC2000 birth cohort
Source: BMJ Paediatr Open. 2024 Aug 30;8(1):e002634. doi: 10.1136/bmjpo-2024-002634 (PMC11367328; doi:10.1136/bmjpo-2024-002634)
Supplement: Supplementary file 2 [file bmjpo-2024-002634.reviewer_comments.pdf]

PEER REVIEW HISTORY

BMJ Paediatrics Open publishes all reviews undertaken for accepted manuscripts. Reviewers are asked to complete a checklist review form and are provided with free text boxes to elaborate on their assessment. These free text comments are reproduced below.

ARTICLE DETAILS

**TITLE (PROVISIONAL)**	Design of the 18-year follow-up of the Danish COPSAC2000 birth cohort
**AUTHORS**	Mølbæk-Engbjerg, Trine<br>Vahman, Nilo<br>Mikkelsen, Marianne<br>Fink, Nadia<br>Christensen, Emil<br>Brustad, Nicklas<br>Sass, Lærke<br>Løvenhøj, Hedda<br>Vinding, Rebecca<br>Schoos, Ann-Marie<br>Stokholm, Jakob<br>Bønnelykke, Klaus<br>Chawes, Bo

VERSION 1 - REVIEW

**REVIEWER NAME**	Anlong Xu
**REVIEWER AFFILIATION**	United Kingdom of Great Britain and Northern Ireland
**REVIEWER CONFLICT OF INTEREST**	No
**DATE REVIEW RETURNED**	16-May-2024

**GENERAL COMMENTS**	This manuscript outlines a promising follow-up visit design for the Danish COPSAC2000 birth cohort. Detailed assessments hold potential for valuable insights into risk factors for atopic diseases, obesity, and neuropsychiatric disorders. However, there are some problems, which must be solved before it is considered for publication. While the potential the assessments is intriguing, the current manuscript lacks details crucial for evaluating its scientific merit.<br>Study Design and Population:<br>1. Including a healthy control group alongside the children born to mothers with asthma would allow researchers to assess the generalizability of the findings and account for potential genetic predisposition.<br>2. Providing estimation of the sample size (n=411) through power calculations would strengthen the study design.<br>3. It is recommended to use a timeline or flowchart to show details of cohort including inclusion/exclusion criteria, sample size in each follow-up visits, and proportions loss of follow-up and when the assessments and tests were conducted.<br>Data Analysis:<br>1. Atopic diseases are basically immunological diseases with complex immuno-metabolic interactions. The authors

	should provide more data and description on immunological or immuno-metabolic assessments.<br>2. While the manuscript describes various assessments, it would benefit from more detailed analyses rather than simply reporting the basic information. Exploring potential associations between these profiles and health outcomes could reveal valuable insights.<br>Deep Analysis:<br>The manuscript focuses heavily on data assessment methods and provides only simple statistical characteristics of the dataset (e.g., means, standard deviations). What are the anticipated contributions of this study to the field? How will the findings inform future research directions? To strengthen the scientific rationale, the authors should consider including a more detailed analysis plan, outlining the specific hypotheses to be tested and the statistical methods to be employed. Deep clinical data is always accompanied by deep analysis.<br>Other Comments:<br>The term “multi-omics” generally indicates integration study of different levels omics data. This study measured mainly clinical features rather than biological sequencing, and it might not be suitable to use “multi-omics” to label it.<br>By addressing these comments, the authors can provide a more comprehensive and scientifically robust foundation for this potentially valuable study.

VERSION 1 – AUTHOR RESPONSE

Formatting Amendments (where applicable):

1) Different Funding Statement

Upon checking your manuscript, I noticed that the Funding Statement in the main documents and the funder listed in the system is different. Kindly update your records and ensure that all data provided in the system should be matched in your main document file.
RESPONSE: This has now been corrected.

2) Missing Grant Number

You have indicated a funder/s for your paper. Please ensure to provide an award/grant number for your funder/s in the submission system. If the funder cannot provide an award/grant number, you can indicate N/A for the award/grant number.

RESPONSE: Award/grant number have now been added where applicable.

3) Different Authors Name Format

The author's name format on the system and the main document file is different. "Ann-Marie Schoos" in the main document while "Schoos, Ann-Marie Malby" in the system. The names indicated in the main text must match the name registered in the ScholarOne submission system.

RESPONSE: This has now been corrected.
4) Supplementary Materials

Please be advised that supplementary materials (appendices, supplementary tables, supplementary figures, etc.) should not be embedded in the main document. These should instead be uploaded as a separate PDF file and cited in the main document in numerical order. E.g. “Supplementary File 1-3” or “Appendix 1-3.”
RESPONSE: This has been corrected as requested.

5) Figure/s should not be embedded
Please remove all your figures in your main document and upload each of them separately under file designation ‘Image' (except tables and please ensure that figures are in better quality or not pixelated when zoomed in). They can be in TIFF, JPG or PNG format. Make sure that they have a resolution of at least 300 dpi and at least 90mm x 90mm of width. Figures in document, excel and PowerPoint format are not acceptable.
RESPONSE: The figures are now uploaded as separate high-resolution files.

Reviewer: 1

Comments to the Author
Thanks for the opportunity to review the manuscript entitled ‘Deep clinical, exposome, and multi-omics assessments of the Danish COPSAC2000 birth cohort: an outline of the 18-year follow-up visit’. This is an outline paper for the 18-year follow-up of the well-known COPSAC2000 birth cohort. The strength of the paper is the long follow-up period, high response rate and the rich information collected from the questionnaires and clinical examinations. The cohort has contributed to our knowledge on pediatric allergic diseases, and are likely to continue with data from this 18-year follow-up.
I only have a few minor suggestions for this manuscript:
C1. Although ‘exposome’ is a popular term, I think it is a bit misleading to include it in the title. By definition the exposome can be a very board term (complementary to the Genome), but it was not described in the paper that information of which specific domain of exposome have been collected or would be investigated. Based on the current manuscript, the collected information regarding environmental exposure was restricted to the indoor environment (pet, environmental tobacco smoking), but are there any other environmental exposure available for the cohort? Have the participants’ addresses been geocoded to be linked to those ambient environment exposures?
RESPONSE: We agree that the term exposome might be misleading, and we have therefore modified the title as follows:
“Design of the 18-year follow-up of the Danish COPSAC$_{2000}$ birth cohort”
We have now added information on the participant's addresses in terms of rural and urban living environments:
Page 14, lines 295-297: *“The cohort exhibited an equal distribution between rural and urban living environments, with 53% residing in rural areas and 47% in urban areas.”*

C2. Same applies for the multi-omics assessment in the title, it is not mentioned in the paper that what omics data have been measured or would be measured for the collected biosamples.
RESPONSE: The title has been modified as follows:
“Design of the 18-year follow-up of the Danish COPSAC$_{2000}$ birth cohort”

The planned omics analysis for the collected biosamples is now detailed in the manuscript as follows:

Page 12, lines 249-256: “***Omics analysis***

We are currently planning the following omics analysis on the biobank materials collected at the 18 year-follow-up visit: 1. Plasma metabolomics, 0-4 hours,

evaluated before and after a nutritional challenge test including 1H-NMR metabolomics measured at eight different times over four hours; 2. Plasma hormones (GIP, GLP1, Insulin, C peptide, and Glucagon), 0-4 hours, evaluated before and after a nutritional challenge test measured at eight different times over four hours; 3 Stool metagenomics (microbiome); 4. Stool metabolomics (Based on NMR), and 5. Stool viromics"

C3. Table 1, page 25 line 37, the BMI categories 'normal weight' and 'overweight' have overlapped on 25 kg/m2.
RESPONSE: This has been corrected to the following:
Table 1, Page 27, line 489: "BMI 18.5-24.99 (%) Normal weight"

C4. A flow chart showing the exact numbers of participants with available questionnaire/clinical examination/biosamples would be useful.
RESPONSE: We have added the following new figures to the manuscript:
FIGURE_1_Flowchart_COPSAC2000.jpeg
FIGURE_3_Flowchart_incl._assessments.jpeg

Reviewer: 2

Comments to the Author
This manuscript outlines a promising follow-up visit design for the Danish COPSAC2000 birth cohort. Detailed assessments hold potential for valuable insights into risk factors for atopic diseases, obesity, and neuropsychiatric disorders. However, there are some problems, which must be solved before it is considered for publication. While the potential assessments are intriguing, the current manuscript lacks details crucial for evaluating its scientific merit.
RESPONSE: Thank you for the valuable suggestions, which we have addressed below in our point-by-point response.

Study Design and Population:
C1. Including a healthy control group alongside the children born to mothers with asthma would allow researchers to assess the generalizability of the findings and account for potential genetic predisposition.
RESPONSE: Thank you for this comment. The COPSAC$_{2000}$ cohort was initially recruited from the Danish National Birth Cohort (DNBC). We have contacted the PI from DNBC and now include baseline characteristics from the 18-year follow-up of DNBC in Table 1 side by side with similar information from COPSAC$_{2000}$ for comparison.
The following has been added to the methods section:

Page 7, lines 135-145, "*The COPSAC$_{2000}$ is a single-center clinical, prospective, mother-child cohort study of 411 individuals born to mothers with asthma who were recruited during pregnancy from the Danish National Birth Cohort (DNBC). The children were included at one month of age and visited the COPSAC research unit at scheduled visits every six months until age 7 and again at ages 12 and 18. Further, the children visited the research unit upon occurrence of any acute airway or skin symptoms. The recruitment and baseline characteristics of COPSAC$_{2000}$ are previously described in detail (1) (see Figures 1-3).*

Initial recruitment into and participation in DNBC, which is a large general population birth cohort has been previously described in details (15). In this study, we compare available and comparable baseline characteristics from a large sample of complete observations from the 18-year follow-up of DNBC with the baseline characteristics of COPSAC$_{2000}$ at age 18."

And this has been added to the *discussion*:

Page 19, lines 396-399, *"A limitation of the study is its external validity because of the high-risk nature of the cohort, which was also predominantly Caucasians. However, comparing the 18-year-olds in the DNBC allows us to compare differences in environmental factors, risk behaviours, obesity, and asthma prevalence in a population-based cohort."*

C2. Providing estimation of the sample size (n=411) through power calculations would strengthen the study design.
RESPONSE: We agree that power calculations are valuable in clinical studies where the effect of an association is tested. However, the $COPSAC_{2000}$ was designed as an observational birth cohort with deep clinical phenotyping to identify and explore early-life exposures and gene-environment interactions in the origins of atopic disease. Thus, no power calculation was performed, but the cohort has provided many novel findings through the years (see e.g.: "25 Years of translational research in the Copenhagen Prospective Studies on Asthma in Childhood (COPSAC)." Bisgaard H, Chawes B, Stokholm J, Mikkelsen M, Schoos AM, Bønnelykke K.J Allergy Clin Immunol. 2023 Mar;151(3):619-633. PMID: 36642652).

C3. It is recommended to use a timeline or flowchart to show details of the cohort including inclusion/exclusion criteria, sample size in each follow-up visits, and proportions loss of follow-up and when the assessments and tests were conducted.
RESPONSE: We have added that information in the following new figures in the manuscript:
FIGURE_1_Flowchart_COPSAC2000.jpeg
FIGURE_3_Flowchart_incl._assessments.jpeg

Data Analysis:
C4. Atopic diseases are basically immunological diseases with complex immuno-metabolic interactions. The authors should provide more data and description on immunological or immuno-metabolic assessments.
RESPONSE: We have now added the following sections to the manuscript.

Page 10, lines 213-218, *"**Immunological assessments***

Blood samples were analysed for multiple immunological outcomes such as C-reactive protein (CRP), IgA, IgG, IgM, IgE, transglutaminase IgA, and IgG, and a broad selection of antibodies such as ANA, ANCA, SR, and CCP.

We also assessed airway immune profiles by quantifying 18 cytokines in upper airway fluid collected with filter paper strips as previously detailed (24). "

Page 16, lines 348-353: *"**Immunological outcomes***

*The mean plasma IgA was 1.49 g/L, with 3.1% of females placed below the normal range vs. 4.5% of males and 2.5% of females placed above the normal range vs. 2.6% for males. IgG had a mean value of 10, with 3.8% of females placed below the normal range vs. 5.8% of males and 0.6% of females placed above the normal range vs. 2.6% of males. The median CRP was 0.74, with an IQR of 1.7 (**Tables S1, S3).**"*

C5. While the manuscript describes various assessments, it would benefit from more detailed analyses rather than simply reporting the basic information. Exploring potential associations between these profiles and health outcomes could reveal valuable insights.

RESPONSE: The purpose of this paper is not to explore the potential associations but rather to outline the hypotheses and planned analyses and provide details of the very thorough 18-year follow-up of our cohort, which will provide other researchers with insight into our methodologies and the depth of the clinical data, environmental exposures, and biosamples.

This purpose is now specified in the introduction of the manuscript as follows:

Page 6, lines 127-132: *"This paper aims to outline the hypotheses and the planned analyses and provide details of the 18-year follow-up visit of the COPSAC$_{2000}$ cohort, including an extensive metabolic and multiorgan assessment and biobanking for omics analyses. This valuable information and material provide a unique possibility for studying risk factors and underlying mechanisms in the origins of atopic disease, obesity, neurodevelopmental disorders, and their overlap."*

Deep Analysis:

C6. The manuscript focuses heavily on data assessment methods and provides only simple statistical characteristics of the dataset (e.g., means, standard deviations). What are the anticipated contributions of this study to the field? How will the findings inform future research directions? To strengthen the scientific rationale, the authors should consider including a more detailed analysis plan, outlining the specific hypotheses to be tested and the statistical methods to be employed. Deep clinical data is always accompanied by deep analysis.

RESPONSE: Thank you for this valuable suggestion. In the introduction, we have now included the overarching major hypotheses to be tested:

Page 6, lines 118-124*: "Our main hypotheses are: 1. Asthma and other chronic non-communicable inflammatory disorders such as obesity and neuropsychiatric disorders are programmed in early life and may share environmental risk factors; 2. Dysregulation of the immune system in early life can lead to chronic inflammatory diseases such as asthma, obesity, and neuropsychiatric disorders; 3. There is an overlap between 18-year-olds with asthma, obesity, and neuropsychiatric disorders; 4. An individual's capacity to metabolise nutrients is associated with risk of asthma, obesity, and neuropsychiatric disorders."*

In the methods section, we have included the following regarding our analysis plan and the statistical methods:

Page 13, lines 270-283: "***Planned analyses***

First, we plan to investigate the association between environmental risk factors and risk behaviours and asthma, obesity, and neuropsychiatric disorders at age 18 using univariate and multivariate logistic regression models. The overlap between asthma, obesity, and neuropsychiatric disorders will be analysed using logistic regression and visualised with Venn diagrams.

Second, we plan to investigate the relationship between the immunological assessments cross-sectionally at age 18 years in relation to asthma, obesity, and neuropsychiatric disorders and longitudinally through childhood using latent class trajectories with multiple assessments from early childhood with random effects incorporated.

Third, we plan to analyse the association between the functional metabolism captured by changes in plasma metabolite levels during the standardised meal challenge using data-driven PCA analysis, time series analysis, and supervised PLS-DA models. In these models, we will integrate plasma hormone levels, gut metagenomics, and stool metabolomics."

In the discussion, we have now elaborated more on the anticipated contributions of this study to the field:

Page 18, lines, 372-381: *"This huge dataset on health and habits, exposures, metabolism, multiorgan assessments, and biosamples for omics profiling from the COPSAC$_{2000}$ birth cohort by age 18 years provides strong data to explore risk factors and metabolic mechanisms behind atopic diseases and other lifestyle-related, non-communicable disorders such as obesity and neuropsychiatric diseases and the commonality between these disorders. The standardised meal challenge is a unique opportunity to study the relationship between an individual's metabolic capacity and risk of asthma, obesity, and neuropsychiatric disorders. This could pave the path for dietary intervention studies to prevent and treat these common disorders. The presentation of this rich data source, biobank material, and methods is also an invitation for collaborative efforts with other cohorts worldwide."*

Other Comments:

C7. The term "multi-omics" generally indicates integration study of different levels omics data. This study measured mainly clinical features rather than biological sequencing, and it might not be suitable to use "multi-omics" to label it.

RESPONSE: We have now removed the term "multi-omics" from the title and manuscript text, and we have also provided more details of the planned omics profiling of the collected biosamples:

Page 12, lines 249-256: "***Omics analysis***

We are currently planning the following omics analysis on the biobank materials collected at the 18 year-follow-up visit: 1. Plasma metabolomics, 0-4 hours, evaluated before and after a nutritional challenge test including 1H-NMR metabolomics measured at eight different times over four hours; 2. Plasma hormones (GIP, GLP1, Insulin, C peptide, and Glucagon), 0-4 hours, evaluated before and after a nutritional challenge test measured at eight different times over four hours; 3 Stool metagenomics (microbiome); 4. Stool metabolomics (Based on NMR), and 5. Stool viromics"

By addressing these comments, the authors can provide a more comprehensive and scientifically robust foundation for this potentially valuable study.
